# A New Scientific Paradigm may be Needed to Finally Develop an HIV Vaccine

**DOI:** 10.3389/fimmu.2015.00124

**Published:** 2015-03-18

**Authors:** José Esparza

**Affiliations:** ^1^Institute of Human Virology, University of Maryland School of Medicine, Baltimore, MD, USA

**Keywords:** HIV-1 vaccines, paradigm shift, non-neutralizing antibodies, vaccine efficacy trials, innovation

## Abstract

The bulk of current HIV vaccine research is conducted within the infectious disease paradigm that has been very successful in developing vaccines against many other viral diseases. Different HIV vaccine concepts, based on the induction of neutralizing antibodies and/or cell mediated immunity, have been developed and clinically tested over the last 30 years, resulting in a few small successes and many disappointments. As new scientific knowledge is obtained, HIV vaccine concepts are constantly modified with the hope that the newly introduced tweaks (or paradigm drifts) will provide the solution to one of the most difficult challenges that modern biomedical research is confronting. Efficacy trials have been critical in guiding HIV vaccine development. However, from the five phase III efficacy trials conducted to date, only one (RV144) resulted in modest efficacy. The results from RV144 were surprising in many ways, including the identified putative correlates of protection (or risk), which did not include neutralizing antibodies or cytotoxic T-cells. The solution to the HIV vaccine challenge may very well come from approaches based on the current paradigm. However, at the same time, out-of-the-paradigm ideas should be systematically explored to complement the current efforts. New mechanisms are needed to identify and support the innovative research that will hopefully accelerate the development of an urgently needed HIV vaccine.

## Kuhn’s Views of Normal Science and Paradigm Change

The bulk of the current scientific activity, at least the one that it is publically funded, is generally conducted within what is known as normal science.

The term “normal science” was proposed by Thomas Kuhn in his 1962 book entitled “*The Structure of Scientific Revolutions*” (1). In his book, Kuhn describes normal science as the “research that it is firmly based upon one or more past scientific achievements, achievements that some particular scientific community acknowledges for a time as supplying the foundation for its further practice.” Those past achievements are generally well-documented in the scientific literature, and serve “to define the legitimate problems and methods of a research field for succeeding generations of practitioners.” In addition, Kuhn coined the term “paradigm” to define the achievements that were “sufficiently unprecedented to attract an enduring group of adherents” and at the same time, “sufficiently open ended to leave all sorts of problems for the redefined group of practitioners to resolve.”

Normal science produces much useful information, but Kuhn proposed that normal science results in little major novelties and it could be argued that it is mostly gap-filling science. Unexpected results that cannot be explained by the current paradigm are frequently ignored or dismissed. When the paradigm is incapable of producing a solution, it enters into a crisis, this leading to a “paradigm shift” which changes the basic assumptions within the ruling theory of science. Then, under the light of a more satisfactory paradigm, some of the previously dismissed observations are acknowledged and understood, and new avenues are opened to conduct new research and to finally solve the problem.

## Current HIV Vaccine Paradigm and Drifts That have Occurred Over the Past 30 Years

Although it is not straightforward to formally define the current HIV vaccine development paradigm, it is not very different from the one that started in the late 1800s, when the germ theory of disease was formulated (2). In summary, the infectious disease vaccine paradigm proposes that different germs cause different diseases, and that the protective adaptive immune responses resulting from the disease can also be stimulated by weakened (attenuated) or killed (inactivated) microorganisms in the form of vaccines. This paradigm has been extremely successful in defining the etiology of major killer diseases of mankind. It has also resulted in the development of vaccines and drugs against many infectious diseases.

However, the infectious disease paradigm, like many other satisfactory paradigms, has gone through minor changes (or paradigm drifts). One change occurred when viruses were described as being structurally different from bacteria, although that did not prevent Louis Pasteur from empirically developing a rabies vaccine, even before viruses were formally discovered (3). In the 1970s retroviruses were widely acknowledged as the cause of cancer and leukemia in animals, but there was strong reluctance to accept the idea that retroviruses could cause infections in humans. When Robert Gallo and collaborators proposed in 1979 that a new retrovirus, the human T-cell lymphoma virus 1 (HTLV-1), causes adult T-cell leukemia (ATL), he found resistance to have the paper accepted for publication, because it represented a departure from the commonly accepted science (4, 5). Fortunately, the common science that was conducted in the 1970s under the “War on Cancer” Program (6) developed the basic tools of retrovirology that also allowed the rapid isolation and characterization of the human immunodeficiency virus (HIV) soon after AIDS was recognized as a new disease in 1981. This is a remarkable example of how research supported to solve a given problem (cancer in this case) can yield unanticipated results in another field (AIDS). What is important is to always keep an open mind.

The decades of the 60s and 70s witnessed the birth of modern molecular biology (7) and rapid progress was made in the understanding of the structure–function relationships in animal viruses. German virologists made many of the pioneering discoveries (8), especially the school of Werner Schäfer (1912–2000) at the Max-Planck-Institut fúr Virusforschung in Tubingen. Studying fowl plague virus (FPV) and other influenza A viruses, Schäfer and collaborators established that the haemaglutinin (HA) serves as a ligand during attachment of the virus cellular receptor and that it was also the immunogen which induces the production of protective neutralizing antibodies in the infected host. Schäfer went on to say in 1963 that “the finding that the immunizing capacity of the fowl plague virus resides in its hemagglutinin shell material led to the proposal to use, instead of inactivated total virus the isolated hemagglutinin of influenza and possibly other myxoviruses as vaccines” (9).

Although we cannot consider this proposed subunit approach as a completely new paradigm, it was obviously a major departure (a paradigm drift) from how viral vaccines were successfully developed until then, based on inactivated or attenuated viruses. It is interesting that two American scientists who were to occupy important roles in the early HIV vaccine effort in the United States, Dani Bolognesi (a virologist from Duke University who became the co-chair of the US government’s AIDS Vaccine Working Group) and Peter Fischinger (who became the first AIDS Coordinator from the US Department of Health and Human Services), had worked with Werner Schäfer in Tubingen, studying the structure–function relationship in animal retroviruses (10). They quickly translated those concepts to the search for an HIV vaccine (11, 12), confirming that the envelope glycoprotein of HIV was sufficient to induce the production of neutralizing antibodies, as it has been shown earlier with the myxoviruses (13).

Since HIV is a very dangerous pathogen, the prospect of using a subunit vaccine was daunting, both in relation to manufacturing issues and because of potential risks to the vaccinated individuals. The response to this challenge was provided by the emerging science of genetic engineering and recombinant DNA technology, which provided the possibility of manufacturing large amounts of viral proteins without actually growing the virus. The most relevant precedent at that time was that of the vaccine against the hepatitis B virus. A highly effective plasma-derived hepatitis B vaccine had been licensed by the US Food and Drug Administration (FDA) in 1981. However, concerns were rapidly raised because the source of the vaccine immunogen was the plasma of individuals who could also be carrying the AIDS virus. The solution came when the surface antigen of the hepatitis B virus was successfully cloned and expressed in yeast, allowing for the manufacturing of a recombinant hepatitis B vaccine, which was licensed in 1986 (14, 15).

Thus, the first wave of HIV vaccine development, based on genetically engineered subunit envelope vaccines (16), was based on several past achievements of normal science: (a) the demonstration that the envelope glycoproteins of the virus are sufficient to induce neutralizing antibodies; (b) the ability to manufacture large amounts of these proteins by genetic engineering techniques; and (c) the successful proof of concept provided by the recombinant hepatitis B vaccine.

This initial vaccine effort also benefited from rapid advances in the molecular biology of HIV that occurred within 5 years after its discovery, including the identification of the major structural proteins of the virus, the cloning and sequencing of the HIV genome, early information on the genetic variability of different virus strains, the description of neutralizing antibodies, and the development of the first non-human primate models (17).

Although in the late 1980s nobody knew for sure how long it would take to develop an HIV vaccine, it is also fair to say that the field was generally optimistic. The prediction was made, and repeated many times since then, that an HIV vaccine would be available within the next 10 years. However, no one knew at that time that HIV/AIDS was much more complex than any other viral disease for which vaccines had been successfully developed (18–21). Nevertheless, phase I clinical trials of HIV envelope vaccines started in the United States in 1988, thus beginning a long history of small successes and big disappointments.

In previous articles, I have discussed in detail the three major approaches that have been explored over the last 30 years in trying to develop an HIV vaccine (17, 22). Although I have described those three waves of vaccine approaches and clinical trials as based on three different paradigms, in fact they only represented allowable tweaks (or drifts) within the overarching infectious disease paradigm. The first wave started around 1984 and it was based on the concept that neutralizing antibodies would be sufficient to confer protection against HIV infection. This led to the development of numerous recombinant envelope-based candidate vaccines that were tested in clinical trials. This first wave came to an end in 2003, with the negative results from two efficacy trials designed to evaluate the protective efficacy of the gp120 vaccines from VaxGen (23, 24). The second wave began with the recognition in the early 2000s of the critical importance of CD8+ T-cell responses in the control of HIV infection, and this led to the development and refinement of live recombinant viral vectors, especially poxvirus and adenovirus vectors, as well as DNA vaccines. This period was formally concluded in 2007 with the unexpected lack of efficacy in the STEP trial, which evaluated a cell-mediated immunity vaccine based on an adenovirus type 5 (Ad5) vector (25). The third wave may have started in 2009 with the modest efficacy obtained in the RV144 trial conducted in Thailand, to evaluate a prime boost combination of an ALVAC vector followed by an envelope glycoprotein (26). This wave, that hopefully will take us to the development of an effective vaccine, should learn from past failures and systematically explore different alternatives, including novel concepts that do not fall within the current paradigm.

However, it is fair to say that the first two waves just described (antibodies and cell-mediated immunity) have not completely ended. Instead, they are constantly revisited when new knowledge is obtained, and numerous adjustments have been made, representing allowable drifts within the current paradigm, with the hope that those changes would eventually led to the solution of the problem.

For example, one of the first conceptual drifts in the antibody approach occurred around 1994 with the realization that laboratory-adapted strains of HIV behave immunologically differently from the primary/clinical isolates (27). That paradigm drift led to the design of more sophisticated envelope immunogens, such as those based on founder/transmitted viruses (28) or those using envelope trimers rather than the gp120 monomers initially used to develop HIV candidate vaccines (29, 30). It is also relevant to mention here that the hope of designing epitope-based HIV vaccines started as early as 1989, when the V3 loop of gp120 was thought to be, and even referred to, as the Principal Neutralization Domain (PND) (31), a vaccine concept that even progressed to phase I clinical trials (32). That early peptide-based HIV vaccine approach was eventually abandoned when it was realized that complex conformational epitopes are important for the induction of neutralizing antibodies (33).

However, a major driver of the more recent approaches in the antibody field has been the need to deal with the immunological variability of HIV strains and clades. The discovery that broadly neutralizing antibodies recognize defined epitopes in the envelope trimer, which has led to a renewed effort to develop epitope-based vaccines guided by structural biology (34, 35), a reductionist approach that claims to have achieved proof of concept with the respiratory syncytial virus (36), but which has been strongly criticized by others (37, 38). The difficulties in designing an epitope-based vaccine capable of eliciting broadly neutralizing antibody responses is compounded by the extensive affinity maturation process that anti-HIV neutralizing antibodies undergo before acquiring the broadly neutralizing characteristics (39). This challenge is being addressed by the use of sequential immunization with different HIV envelope immunogens designed to guide the evolution of the antibody, triggering the selection and expansion of germline precursor and intermediate memory B cells to recapitulate B cell ontogenies associated with the maturation of a broadly neutralizing antibody response (40, 41), a concept that had been proposed several years before (42). Others, perhaps more practical approaches, have been proposed to develop a globally relevant HIV vaccine capable of protecting against a variety of strains and clades (43), including the use of mosaic immunogens (29, 44).

On the other hand, most of the paradigm drifts in the field of cell-mediated HIV vaccines have focused on the use of different vectors, or prime-boost approaches, with the object of eliciting stronger and more functional CD8+ responses to selected HIV proteins (45–47). Perhaps, the most significant paradigm drift in the cell-mediated immunity field is represented by the report that the early control elicited by a simian immunodeficiency virus (SIV) protein-expressing rhesus cytomegalovirus (RhCMV) vectors (48) was due to SIV-specific CD8+ effector memory T cells that recognize unusual, diverse, and highly promiscuous epitopes, including dominant responses to epitopes restricted by class II major histocompatibility complex (MHC) molecules (49–51).

## Efficacy Trials have been Instrumental in Drifting Paradigms and Advancing Vaccine Research

In the absence of predictive animal models, or of known immune correlates of protection, the only approach to assess the protective efficacy of any HIV vaccine concept is by conducting large scale efficacy trials of the candidate vaccines considered to be the most promising. However, what is considered to be the “most promising” candidate vaccines usually is in the eyes of the beholder. Since efficacy trials are complex and expensive, it is widely recognized that a decision to proceed to phase III trials needs to be based on the best science available, and this responsibility should not be taken lightly. On the other hand, the urgent public health need of an HIV vaccine should be considered in order not to delay those important decisions (52).

A case in point is the RV144 efficacy trial conducted in Thailand between 2003 and 2009. The trial was strongly opposed by a group of respected scientists, who were not convinced of its scientific merits (53). Anyway, the trial went ahead and it was conducted almost totally ignored by the scientific community. When the announcement was made in 2009 that the RV144 trial showed modest efficacy (26), it came as a surprise and the results were initially received with skepticism. After all, the results contradicted what was commonly accepted at that time, namely that the most likely protection that a vaccine could provide was against virus load and not against virus acquisition. In addition, the identified immune correlates of protection were not the usual suspects (neutralizing antibodies or CD8+ T cells). Instead, a still to be better defined non-neutralizing antibody response to the V1–V2 loops of gp120 was found to be the strongest correlate of protection (54). Subsequent laboratory studies have strengthened the conviction that the protective efficacy observed in RV144 is true, and have identified IgG3 antibodies as an additional potential correlate of protection (55).

The results from the RV144 trial have stimulated new research on antibody functions other than neutralization, such as antibody-dependent cell-mediated cytotoxicity (ADCC) (56, 57), which is slowly becoming part of the accepted normal science in HIV vaccine research, side by side with the better understood neutralizing antibodies. The newly acquired respectability of ADCC should help understanding the earlier results reported by Robert Gallos’s team in 2007 using subunit immunogens designed to raise humoral responses against CD4-induced (CD4i) epitopes (21, 58, 59). Even earlier, in 2005, ADCC was reported as an immune correlate of protection against SIV in non-human primate protection (60). However, the results from those experiments were received with skepticism or even indifference, because at that time our minds were not prepared to think outside of the box regarding antibody functions. The classical neutralizing antibodies are probably the most important mechanism of protection against HIV, as is the case with most viral vaccines, but the potential role of ADCC and of other antibody functions should not be dismissed *a priori* (61, 62).

From the five efficacy trials of HIV vaccines that have been completed in the last 10 years, only RV144 showed efficacy, albeit modest (17, 23–26, 63). To build on the success of the RV144 trial, a group of organizations established the Pox-Protein Public-Private Partnership (P5) to evaluate potentially improved pox-protein vaccines to determine if they might provide significant public health benefit, with follow-up clinical studies using improved vaccine regimens being planned in southern Africa and Thailand (64). A very important objective of the P5 is to validate the hypothesis that in the RV144 trial, antibodies directed against the V1–V2 loops may have contributed to protection against HIV-1 infection, whereas high levels of envelope-specific IgA antibodies may have mitigated the effects of protective antibodies (54). What is now critical is to develop strict and credible go/no-go criteria to determine if the potentially improved vaccines should move from phase I clinical trials to large scale efficacy evaluation, including the ability to test the hypotheses generated by the RV144 trial. In making that decision, it is important to keep in mind that the RV144 trial was conducted in Thailand in a population with relatively low risk behavior and an annual HIV incidence of approximately 0.2% (65, 66), and that the proposed P5 trials are planned to be conducted in populations with annual HIV incidences in the order of 3–9%. One could argue that the vaccines to be tested by the P5 collaborators should be proportionally improved, considering the stronger force of infection in the proposed new testing population.

Since phase III efficacy trials are large and expensive, every effort should be made to obtain pre-clinical and early clinical evidence to justify such a decision. Although non-human primate protection experiments are instructive, and a positive result would add confidence to a decision to move to efficacy evaluation, they are not necessarily considered as predictive of results in humans. An alternative, or rather complementary approach to select candidate vaccines for further evaluation, has been proposed by testing candidate vaccines in a handful of human volunteers whose immune system is intensively interrogated in the search for clues that may suggest the induction of protective immunity. These small trials, referred by some as “Experimental Medicine” (EM) trials, could be very valuable for vaccines for which we have known immune correlates of protection (67), but they present a challenge for HIV. However, we can imagine that envelope immunogens designed to induce broadly neutralizing antibodies, including approaches that guide their maturation, could be tested in EM trials (40). Likewise, human CMV vectors could be tested in EM trials to assess if they recapitulate in humans the potentially protective immune responses that have been identified in rhesus monkeys (49).

Perhaps, the identification of a single protective epitope or of a single immune correlate of protection is an illusion derived from our desire to reduce complex biological phenomena to simple explanations and approaches. Rather than thinking about just one individual immune correlate, we should seriously consider that protection is associated with a more complex immunological signature of immune responses. Fourteen years ago, Neil Nathanson and Bonnie Mathieson, from the Office of AIDS Research of the US National Institutes of Health, speculated that a “possible explanation for the inconsistent conclusions from studies of SIV and SHIV models is that protection does not correlate with any single immune response but is conferred by a barrier created by the sum of several immune defenses” (68). In fact, the coordinated activity of multiple antibody functions has been recently suggested as the mechanism of protection in a proposed globally relevant HIV-1 mosaic vaccine (44).

## Can We Identify Innovation When We See It?

There is no doubt that many of the paradigm drifts introduced over the years to the original antibody and cell-mediated immunity concepts have been very innovative, although they have not represented significant shifts of the prevailing paradigm. Scientists have stubbornly pursued their belief that the current paradigm, once it is appropriately modified, will provide the solution. Resilience is important for the progress of science. However, and paraphrasing the immunologist and Nobel Prize winner Peter Medawar, “the intensity of the conviction that a hypothesis is true has no bearing on whether it is true or not” (69). Nevertheless, Medawar emphasized that the strength of the scientists’ convictions is important if only because that conviction provides the necessary incentive to conduct the research to find out if the hypothesis is correct.

The scientific community is generally open to accept innovation when it falls within the accepted paradigms of normal science. However, the same community is often reluctant to accept ideas that fall outside of the paradigm. The possible reasons for this attitude are that most of those out-of-the paradigm ideas are: (a) sometimes proposed without much preliminary data, (b) not supported by a community of peers and, (c) in many cases, cannot stand up to critical scrutiny or to experimental verification.

The question that we are now trying to answer is if after 30 years of intense work, the current paradigm to develop an HIV vaccine is entering into a crisis, thus requiring a paradigm shift. Is it sufficient to go back to the same drawing board every time we experience a major failure? (70), or should we explore more systematically completely new avenues of research? Perhaps the nature of HIV and AIDS, which significantly differ from other viral diseases for which vaccines have been developed, provides the explanation for the repeated failures in our attempts to stick to the current approaches (18–20). Perhaps, the current paradigm is not appropriate to develop vaccines for a virus that profoundly affects the immune system of the host and that uses many different mechanisms to escape what otherwise could be protective immune responses.

A related question is if we have in place the appropriate mechanisms to identify and support the highly innovative science that could allow for a paradigm shift? The current peer review system, which has been extremely efficient in protecting the quality of normal science, may not be the best system to stimulate out-of-the-paradigm research, with innovative concepts placed at high risk of being suppressed (71, 72). This challenge has been recognized by different institutions, leading to the creation of special initiatives to stimulate innovation on HIV vaccine research. These include the Innovation Grant Program for Approaches in HIV Vaccine Research created by in 1997 by the so-called Baltimore Committee of the National Institute of Allergy and Infectious Diseases (73, 74), the Grand Challenge on Global Health Program from the Bill & Melinda Gates Foundation (75), and the Innovation Fund from the International AIDS Vaccine Initiative (IAVI) (76). Unfortunately, those programs, that have (or had) their own mechanisms to select and monitor projects, by and large have failed to spur the necessary innovation. The problem has been that it is very difficult to predict what innovative projects will work, or even to suggest any specific areas of exploration. However, what it is possible is to formally establish innovative processes and mechanisms to support such research.

In 2007, the Wellcome Trust and the Bill & Melinda Gates Foundation convened a meeting in London to discuss the need to bring additional innovation to HIV vaccine research. The group recognized that innovative proposals are high-risk, that peer review is conservative and risk adverse, and that peer-review can’t deal with proposals that challenge accepted thinking. In considering innovative research, the group recommended that funders consider using broad-minded people who look at impact and at the big picture, ask thoughtful questions, and give applicants a chance to reply (77).

It is fair to say that that innovative HIV vaccine concepts that are not part of the mainstream thinking are regularly published. Very often those articles are initially rejected in more prestigious journals, and the authors usually struggle to secure the funds needed to advance the research and to eventually confirm and expand, or to refute the original observations. For instances, in 2012 Jean-Marie Andrieu and collaborators reported that oral immunization of chinese Rhesus macaques with a combination of *Lactobacilus plantarum* and inactivated SIV provided strong protection against subsequent infection with the virus (78, 79). More surprisingly, the observed protection did not correlate with any known adaptive immune response, but instead it correlated with CD8+ regulatory T cells that seemed to mediate a tolerogenic mechanism that falls outside of the current paradigm. Not surprisingly, the authors experienced difficulties in getting the paper accepted by different journals, and the results were received with a great deal of skepticism. Fortunately, the Bill & Melinda Gates Foundation was able to support an independent confirmation of those observations. At least in this case, a potentially game-changing idea was not dismissed *a priori*, and the results of the potentially confirmatory study will be available at the end of 2015.

Although it is beyond my individual predictive abilities to identify what could be the most promising out-of-the-paradigm concepts, a few additional examples could be listed for further exploration. One is the use of HIV-1 gp-41 subunit virosomes, which have been shown to be protective in non-human primate models, with protection correlating with mucosal antibodies rather than with circulating neutralizing antibodies (80–83). Another vaccine concept, also based on a gp41 peptide, was reported to protected CD+ T cells from lysis by natural killer cells, without having any protective effect against the infection *per se* (84, 85). Finally, although whole inactivated vaccines were extensively tested in the past in animal models, with negative results, perhaps it is not unreasonable to revisit this concept using current experimental approaches, including low-dose repeated challenges (86).

The barriers to accept new concepts or paradigms cannot be underestimated. This was well understood by the German theoretical physicist Max Planck when he said that “a new scientific truth does not triumph by convincing its opponent and making them see the light, but rather because its opponents eventually die, and a new generation grows up that is familiar with it” (from Max Planck’ Scientific Autobiography, cited by Kuhn) (1).

## Do We Need a New Scientific Paradigm to Finally Develop an HIV Vaccine?

The answer to that question is: perhaps. The important point is that, while we continue pursuing the current approaches, we should also actively explore new avenues, leaving no stone unturned in our search for an HIV vaccine (87).

After all, 30 years of intense HIV vaccine research has not resulted in a practical effective vaccine, although such vaccine is sorely needed to bring the HIV epidemic under control (52). In order to accelerate the development of an HIV vaccine, we recently proposed a number of actions, including the suggestion to establish a program of truly innovative research with protected funding to explore out-of-the-paradigm approaches, perhaps allocating to this program not less than 10% of the total HIV vaccine investment (22). Innovative research, especially out of the paradigm frame, needs to be supported by an innovation ecosystem which should include, not only the innovative scientists, but also an enlightened leadership in the field, the appropriate mechanism for the selection of projects and, perhaps more importantly, a supportive scientific community (88).

HIV vaccine research needs to continue with the sense of urgency that the severity of the AIDS pandemic is imposing on us. We constantly need to keep in mind that the objective of our research is not only the acquisition of new knowledge, but the developing of a practical solution for one of the worse public health problems of our time.

## Conflict of Interest Statement

The author declares that the research was conducted in the absence of any commercial or financial relationship that could be construed as a potential conflict of interest.
